# Society for the Relief of Widows and Orphans of Medical Men

**Published:** 1810-12

**Authors:** 


### *To the Editors of the Medical and Physical Journal.*

### *Society for the Relief of Widows and Orphans of Medical Men.*

GENTLEMEN,

I DO not recollect that any notice has been taken in the Medical and Physical Journal, of the meritorious Society for the "Relief of the Widows and Orphans of Medical Men in London and its Vicinity," which was established in the year 1788, and to which, it is to be regretted, that comparatively few of the Profession belong. The number of members being now only 282, though there are upwards of 700 members of the profession, residing within the limits, to which this society extends the advantages, that may be derived from belonging to it.

In the Address to the Public, with which this society began its institution, it is said, "It is astonishing that medical men, in this metropolis, should have been so long inattentive to the benefits which their families might derive in case of distress, from a well-regulated establishment of this kind. The opinion of the world discountenancing celibacy in them, is one principal cause why there is proportionally a greater number married

 in in this than any other class of men. This circumstance, with the many obstacles which oppose a productive establishment in business, especially when the practitioners are young, often prevents them from making suitable provision for their families in case of death. And if to these circumstances it be added, that they are from the nature of their profession particularly exposed to contagion, and other causes of disease, more arguments need not be urged to enforce the necessity of promoting some scheme to alleviate the distress of the families of medical men, when death deprives them of a husband or father."

The truth and justice of these observations have been fully confirmed by what has occurred among the members of this society. Since its establishment in 1788, 97 deaths have happened, which have occasioned applications for relief from 21 widows and families of the deceased; so that though, in the list of deaths are the names of many men who entered into the society solely from philanthropic motives, and without any view of future advantage to their own families, yet it appears that the families of more than one in five of the members have been left in circumstances of penury and distress.

To declaim upon the vicissitudes of life is easy, for they are of daily and hourly occurrence; and there can never be a want of materials to him who wishes to enlarge upon this subject. Among the widows and orphans who have been compelled to seek relief from the beneficence of this society, many melancholy examples of this nature might be collected. Men who have entered into professional life with the fairest prospect of success, whose wives and children have been accustomed to a degree of gentility and elegance, and who have reasonably expected a continuance of comfortable enjoyment, have suddenly been arrested by death, and their families have only been saved from the parish workhouse by the assistance of this society.

And yet assistance has been afforded to those only, who were left in a state of the most miserable penury; for till of late, the widows and orphans of no member were entitled to receive assistance, if from the effects of the deceased, the poor pittance of thirty pounds a year could be scraped together for the widow, and ten pounds a year for each of the children.

Happily the society is now in a condition to be more liberal; but with the prospect before it of a great accession of claimants in the course of years, it is not possible to do so much present good as it wishes. Its funds must still be economized with the most scrupulous regard to future calls upon them; for it has been calculated that in less than thirty years, it will be obliged to distribute, annually, upwards of *one thousand pounds*, among the widows and orphans, even supposing that the average allowance to each family should be only 25l. per annum.

This

This society possesses advantages which do not belong to common benefit societies; for as there are among the members, a great many who can never want any assistance from it, so there is more to be distributed among those who really do require it, and the fine of admission and annual contribution is consequently very much smaller than could otherwise be possible. In fact the indigent members of this society, by the payment of *only two guineas* a year, are entitled to greater advantages than they could enjoy in other societies of the same kind, by the payment of a much more considerable annual contribution.

That a judgment may be formed of the advantages which have resulted to some of the widows and orphans of members, I will just mention that the families of twelve members who altogether paid only 125 guineas in contributions, have received collectively, the sum of 2340l., and the majority of these twelve families are still receiving the assistance of the society. Among these are the orphan children of a surgeon, who was only one month a member, and who only paid two guineas, yet to his family has been paid by this society the sum of 390l.

| | *l.* | *s.* | *d.* | | *l.* | *s.* | *d.* |
|---|---|---|---|---|---|---|---|
| A Physician paid . | 3 | 3 | 0 | His widow has received | 390 | 10 | 0 |
| An Apothecary paid | 5 | 5 | 0 | His widow has received | 252 | 10 | 0 |
| A surgeon paid . . | 7 | 7 | 0 | His widow has received | 135 | 10 | 0 |

That a society so well constituted, and so abundantly useful, should be enabled to extend its powers of doing good, must be the wish of all men; and I am persuaded that its principles and its practice require only to be more known, in order to render the number of its members much greater.

There are three classes of medical men in London and its environs, for whom I cannot imagine any excuse, if they do not become associates in this excellent institution.

1st. Those who know that their circumstances are unfortunately so low, as to allow very little chance of their leaving their families any adequate support. For the small sum of *two guineas* annually, and a very moderate fine on admission, they may become joint proprietors in a fund which at present amounts to nearly 18,000l., and which is progressively increasing. And they will have the farther satisfaction of knowing that the distresses of their widows and children will come under the cognizance not of hard and unfeeling parish officers, but of their professional brethren, who best know how to appreciate their losses and misfortunes.

2dly. Those who have little reason to dread the distress of the foregoing class, but who, notwithstanding, may, from various accidents, be thrown out of the course in which they are at present present doing well. These partly from prudential, partly from philanthropic motives, ought to inscribe their names in the lists of this society.

3dly. Those, more especially, ought to belong to this Society, who have already attained such a rank in the profession, as to place them out of the reach of the misfortunes, which too often assail their more indigent brethren. Let those who have risen to fame and fortune by the practice of medicine, shew their gratitude for the emoluments that profession has conferred on them, by sparing a trifle from their superfluities towards the relief of the unhappy women and children, who have no husband to succour, no father to support them. It must give pleasure to every liberal-minded man to see his own profession respectable and respected. Let him then contribute to the means of adding to its respectability, by preserving from misery, from sorrow, and perhaps from vice, the widows and children of his professional brethren. Thus will he prove an honor to himself, and advantage to his friends, and a benefit to the community. Sibi ipsi honori, et amicis utilitati, et Reipublicæ emolumento esse possit.—*Cicero.*

*Curzon-street, Nov.* 7, 1801. S. M.

---

